# Ad hoc instrumentation methods in ecological studies produce highly biased temperature measurements

**DOI:** 10.1002/ece3.3499

**Published:** 2017-10-20

**Authors:** Adam J. Terando, Elsa Youngsteadt, Emily K. Meineke, Sara G. Prado

**Affiliations:** ^1^ Department of Interior Southeast Climate Science Center US Geological Survey Raleigh NC USA; ^2^ Department of Applied Ecology North Carolina State University Raleigh NC USA; ^3^ Department of Entomology and Plant Pathology North Carolina State University Raleigh NC USA; ^4^ Department of Organismic and Evolutionary Biology Harvard University Herbaria Cambridge MA USA; ^5^ North Carolina Cooperative Fish and Wildlife Research Unit Department of Applied Ecology North Carolina State University Raleigh NC USA

**Keywords:** air temperature, climate change, data logger, HOBO, iButton, radiation shield

## Abstract

In light of global climate change, ecological studies increasingly address effects of temperature on organisms and ecosystems. To measure air temperature at biologically relevant scales in the field, ecologists often use small, portable temperature sensors. Sensors must be shielded from solar radiation to provide accurate temperature measurements, but our review of 18 years of ecological literature indicates that shielding practices vary across studies (when reported at all), and that ecologists often invent and construct ad hoc radiation shields without testing their efficacy. We performed two field experiments to examine the accuracy of temperature observations from three commonly used portable data loggers (HOBO Pro, HOBO Pendant, and iButton hygrochron) housed in manufactured Gill shields or ad hoc, custom‐fabricated shields constructed from everyday materials such as plastic cups. We installed this sensor array (five replicates of 11 sensor‐shield combinations) at weather stations located in open and forested sites. HOBO Pro sensors with Gill shields were the most accurate devices, with a mean absolute error of 0.2°C relative to weather stations at each site. Error in ad hoc shield treatments ranged from 0.8 to 3.0°C, with the largest errors at the open site. We then deployed one replicate of each sensor‐shield combination at five sites that varied in the amount of urban impervious surface cover, which presents a further shielding challenge. Bias in sensors paired with ad hoc shields increased by up to 0.7°C for every 10% increase in impervious surface. Our results indicate that, due to variable shielding practices, the ecological literature likely includes highly biased temperature data that cannot be compared directly across studies. If left unaddressed, these errors will hinder efforts to predict biological responses to climate change. We call for greater standardization in how temperature data are recorded in the field, handled in analyses, and reported in publications.

## 1 INTRODUCTION

Global surface temperatures have increased an average of 0.85°C since 1880 (Hartmann et al., 2013), resulting in profound changes in phenology (Anderson, Inouye, McKinney, Colautti, & Mitchell‐Olds, 2012; Walther et al., 2002), abundances (Parmesan, 2006), and distributions (Chen, Hill, Ohlemüller, Roy, & Thomas, 2011; Moritz & Agudo, 2013) of species worldwide. Yet, large uncertainties remain about the ecological consequences of recent warming. The construction of accurate, mechanistic models of biological sensitivity to climate change will be a critical part of management efforts to prevent global biodiversity loss (Urban et al., 2016). These models require accurate and precise estimates of temperature at the scale of relevant biological processes.

Permanent, stationary weather stations are currently the gold standard for monitoring air temperatures in the field (Diamond et al., 2014; Forister & Shapiro, 2003; Marra, Francis, Mulvihill, & Moore, 2005; Rundel, Graham, Allen, Fisher, & Harmon, 2009). However, these instruments are sparsely distributed and are often sited in flat, open areas. In contrast, biological processes, and therefore terrestrial field ecology studies, are more likely to operate at finer spatial scales. At these scales, local environmental variation more strongly affects air temperature (Fridley, 2009; Potter, Woods, & Pincebourde, 2013), which in turn affects numerous biological and ecological processes (Chen et al., 1999). In response to the need for these microclimatic data, ecologists have increasingly turned to small and inexpensive environmental sensors (also known as data loggers) that can be quickly deployed and simultaneously record observations at high densities in the field. These devices capture the local variance structure of air temperature, improving ecologists’ ability to make inferences about its effect on the biota of interest. It is worth noting that other thermal parameters, such as operative temperature or habitat surface temperatures, may be the most biologically relevant measures for some research questions (Bakken, 1992; Huey, Peterson, Arnold, & Porter, 1989). We focus here on air temperature because it has a long historical record, is widely used, and has potential to be consistently deployed in ways that allow comparison between studies conducted in different habitats.

Temperature sensors vary in accuracy, precision, and price, which can make choosing among them difficult. In addition to these internal differences, sensors are sensitive to solar radiation, which can result in significant biases in recorded observations during periods of direct sunlight and high temperatures (Holden, Klene, Keefe, & Moisen, 2013; Hubbart, Link, Campbell, & Cobos, 2005). Radiation shields can buffer these inaccuracies (Holden et al., 2013; Hubbart, 2011) but are not available for many inexpensive sensors and are marketed as an optional purchase with more expensive ones. As a cost‐effective solution, ecologists have developed several types of radiation shields using inexpensive everyday materials. These custom‐fabricated shields seemingly obviate the need to purchase expensive manufactured shields, allowing ecologists to take full advantage of the low‐cost temperature sensors.

And yet, we are aware of only a small number of studies wherein the efficacy of custom‐fabricated radiation shields for use with low‐cost data loggers is assessed through systematic comparison to manufactured shields or permanent weather stations (Ashcroft & Gollan, 2013; Cheung, Levermore, & Watkins, 2010; Holden et al., 2013; Hubbart, 2011; Hubbart et al., 2005; Lundquist & Huggett, 2008; Tarara & Hoheisel, 2007). These studies, none of which are in ecology‐focused publications, have generally concluded that sensors housed in custom‐fabricated shields provide acceptable accuracy, with mean biases of <1°C relative to the chosen standard (i.e., a weather station or a sensor housed in a manufactured shield) but results vary across environments. For example, a shield constructed of two modified funnels performed nearly as well as manufactured shields in a greenhouse (Hubbart, 2011) but yielded a bias of up to 8°C in field tests (Holden et al., 2013). Such differences among custom‐fabricated shields cast doubt on the accuracy and comparability of temperature data reported in studies that rely on different (and often untested) methods. Moreover, given the cost advantages of the least expensive temperature sensors, these errors could rapidly proliferate if standardized methods to minimize biases are not developed and adopted in field ecology, significantly hindering our ability to accurately predict species responses and sensitivities to climate change.

Here, we assess current trends in the use and shielding of portable temperature sensors by sampling 18 years of ecological literature. We then present the results of an experiment designed to test the accuracy of the most commonly used small, portable temperature sensors across three environmental settings where field ecology is often carried out: open fields, closed‐canopy temperate forests, and urbanized areas. The first two sites were colocated with permanent weather stations that included high‐quality temperature sensors, while the urban sites spanned a gradient of impervious surface cover that reveal how observation biases can vary across habitats in a typical field study. We applied multiple treatment combinations consisting of manufactured and custom‐fabricated radiation shields. Our goal was to provide a much‐needed advance in the development of standardized methods for accurately measuring and monitoring air temperature, when using inexpensive sensors in field ecology and global change studies.

## 2 MATERIALS AND METHODS

### 2.1 Assessment of current practice in ecology

We conducted a literature search to determine whether the use of small, portable temperature sensors in ecology has become more common over the past 18 years. We focused our review on HOBOs (Onset Computer Corporation, Bourne, MA) and iButtons (Maxim Integrated, San Jose, CA) as, anecdotally, these are commonly used sensors in field ecology. Although “HOBOs” include a variety of portable environmental sensors, we used the broad search for “HOBO” because model names and numbers are not consistently reported in the literature (see the Supplementary Information for complete list of HOBOs listed in the papers examined). We then assessed a subset of the recovered papers to determine how ecologists were using these sensors, and, in the case of air temperature measurements, whether and how sensors were shielded from direct and diffuse solar radiation. Because a Web of Science search returned few relevant results for the names of common sensors (“iButton” and “HOBO”), we selected 20 journals to search directly. To identify target journals, we used the ISI Journal Citation Report for the category “ecology” and considered all journals with a 5‐year impact factor >3.5. We then excluded journals that publish primarily reviews, the scope of which did not include field ecology, and those that yielded no search results for “iButton” or “HOBO.” The 20 journals used are listed in Table S1.

To assess how the frequency of iButton and HOBO use has changed over time, we used each journal's own website search function to search separately for key words “iButton” and “HOBO.” We scanned the results to confirm relevance, for example, that “HOBO” referred to an environmental sensor and not a genetic element, and that all results were original research contributions and not review articles. We recorded the number of papers published using each sensor type in each year from 1998 through 2015. We chose 1998 as the starting year because it was the first year of publication or online archiving of relevant journals such as *Ecology Letters*,* Diversity and Distributions*, and *Ecosystems*. To avoid inflating the number of studies in 2015, we excluded papers that were published online in 2015 pending assignment to a 2016 issue. We performed all searches in February 2016.

To examine the details of how ecologists use and shield iButtons and HOBOs, we examined up to 25 papers per journal from the “iButton” searches. This resulted in a sample of 170 papers, representing 100% of studies in the designated time period in all journals except *Oecologia*, from which we randomly selected a subset of 25 of the 40 papers available. As the “HOBO” search returned almost three times as many records compared to the iButton search, we randomly selected a subset of papers from each journal to equal the number of iButton papers from the same journal. Hence, we also examined a total of 170 papers that mentioned HOBO sensors (1–25 per journal). Six papers were shared between the iButton and HOBO groups; the total number of papers examined was 334 (see Supporting Information). For each paper, we recorded sensor identities and environmental parameters measured (e.g., soil temperature, air temperature, and water temperature). For those that measured air temperature, we further recorded any details or citations about how the sensor was protected from solar radiation.

### 2.2 Field experiment: temperature sensors and radiation shields

**Figure 1:**
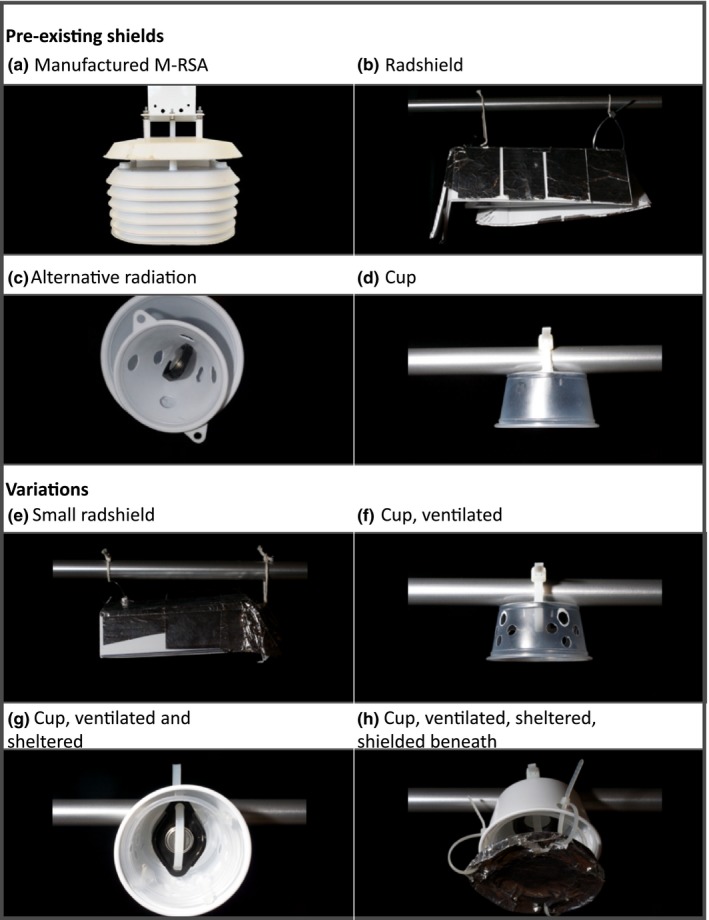
Custom‐fabricated radiation shields tested in the field. (a–d) are radiation shields previously used in published research papers. (a) A manufactured radiation shield, also referred to as a “gill shield” (Onset Computer Corp., Bourne, Massachusetts, part MRSA). (b,c) Custom‐fabricated radiation shields tested by Holden et al. (2013) and Hubbart (2011), respectively. (d) A custom‐fabricated shield used in a field study (Carper et al., 2014; Meineke et al., 2013). (e–h) are radiation shields created for testing in this study. (e) A smaller version of (b). (f) A modification of (d) with holes to allow airflow. (g) A modification of (c) using a larger white cup. (h) A modification of (g) with a shield placed below the sensor. Construction details are provided in the Fig. S1

To assess the accuracy of commonly used environmental sensors, we conducted a field experiment using a total of twelve temperature sensor‐radiation‐shield combinations to compare to permanent weather stations. We used three types of temperature sensors: iButton (DS 1923 Hygrochron, Maxim Integrated, San Jose, CA), HOBO Pendant (UA‐001‐08, Onset Computer Corporation, Bourne, MA), and HOBO Pro (U23‐001 Pro v2, Onset Computer Corporation; Table S2). The HOBO Pro is a self‐contained data logger with an attached thermistor‐based temperature sensor; the HOBO Pendant is a smaller and lower‐cost thermistor‐based data logger, and the iButton hygrochron is a low‐cost data logger with a silicon‐based internal temperature sensor. We assigned each sensor model to an unshielded treatment and at least one (up to seven) shielded treatments (Figure 1).

Specifically, we assigned HOBO Pro sensors to only one shielded treatment, the manufacturer‐recommended M‐RSA naturally ventilated multiplate solar radiation shield (also known as a “Gill” shield, (Gill, 1979); Figure 1a). We also subjected HOBO Pendants to only one shielded treatment, the custom‐fabricated Radshield of Holden et al. (2013) (Figure 1b). We subjected iButtons to the seven custom‐fabricated shield treatments shown in Figure 1 (no manufacturer‐recommended radiation shield exists for the iButtons). These include three previously described shields and four original variations. The previously described shields include (i) the Radshield of Holden et al. (2013) (Figure 1b); (ii) the Alternative radiation shield of Hubbart (2011) (Figure 1c); and (iii) a translucent plastic cup, as described by Meineke, Dunn, Sexton, and Frank (2013) and Carper, Adler, Warren, and Irwin (2014) (Figure 1d). All were constructed to the best of our abilities, based on our understanding of the methods and materials used. However, minor deviations in our attempted replication of the authors’ methods may affect any reported biases. The first of the original variations was a smaller radiation shield similar to that of Holden et al. (2013) with the dimensions reduced by 50% (Figure 1e). We also tested three variations of the plastic cup design that incorporated (i) ventilation (Figure 1f), (ii) ventilation and a more reflective, white outer cup (Figure 1g), and (iii) ventilation, white outer cup, and a basal foil shield to protect the sensor from radiation reflected from the ground (Figure 1h). Details of shield materials and dimensions are included in Fig. S1. iButtons in all treatments were mounted using iButton wall mounts (DS9093S, Maxim Integrated) with twist ties or cable ties. We tested more iButton treatments than HOBO treatments because iButtons are smaller, less expensive, and less conspicuous than either HOBO sensor, often making them more attractive for large‐scale studies, and in areas where theft or esthetics are an issue. Many of the tested iButton shields were too small to accommodate HOBO sensors.

### 2.3 Sensor accuracy in exposed and forested locations

To examine the accuracy of unshielded and differently shielded temperature sensors, we used 60 sensors, comprising five replicates of each of the 12 sensor‐shield combinations (Table S3). All sensors were programmed to record temperature synchronously every 30 min (on the hour and half‐hour) and then were deployed to record temperatures at two sites that contain permanent weather stations with calibrated temperature sensors. The first weather station (referred to as Lake Wheeler), part of the North Carolina Environment and Climate Observing Network (ECONet), is located in a fully exposed rural location near Raleigh, NC (35.728°N, 78.680°W). This station is outfitted with a VAISALA platinum resistance air temperature sensor (HMP45C) mounted inside a wind‐aspirated multiplate radiation shield (Fig. S2a). Air temperature was logged at this site for the 16‐day period covering 6–21 August 2015 in typical summer conditions for the southeastern United States. We then placed the 60 sensors in a rural forested location from 21 to 28 August 2015, at a Remote Assessment of Forest Ecosystem Stress (RAFES) network fixed weather station (referred to as Duke Forest) near Durham, NC (35.98°N, −79.09°W; Fig. S2b), which also uses a naturally aspirated multiplate radiation shield. Sensors were randomly placed on 1–2 meter‐long 25 mm × 51 mm wood boards, which were passed through the instrument tower at each station, placing the sensors at the same height as the permanent wind‐aspirated temperature sensors, approximately 2 m above the ground. The fixed weather stations recorded temperature every minute (Lake Wheeler) or every hour (Duke Forest), and we obtained these data for the time periods that our sensors were installed at each station.

### 2.4 Variation in sensor performance along an impervious surface gradient

To determine how sensor accuracy varied across field conditions, we placed sensors at five sites along an impervious surface gradient, which allowed us to evaluate the effect of variation in upward‐directed radiation on sensor accuracy. At each site, we selected a focal tree and suspended 12 sensors, one per sensor/shield combination, on a branch 2–3 m above ground. Sensors continued to record synchronously every half‐hour and were on site 2–9 September 2015. We measured impervious groundcover within 100 m of the focal tree using ArcMap version 10.3.1 (ESRI, Redlands, CA), with a 1 m resolution impervious surface map of Raleigh, NC, obtained from the Wake County GIS Map Services website (http://www.wakegov.com/gis/services/pages/data.aspx). The impervious surface sites included a near‐urban forested site (0% impervious surface), a suburban residential backyard (20%), an urban residential front yard (31%), a street‐side lawn (41%), and a parking lot (46%).

### 2.5 Data analysis

All statistical analyses were conducted using R version 3.3 (R Core Team, 2016). We used two error metrics to assess overall bias and accuracy of the sensor/shield treatments in sunny and shaded conditions. First, we calculated the average bias of each sensor treatment in relation to the two permanent weather stations:

$$\text{bias}=\frac{1}{n}\sum_{i=1}^{n}\left(s_{i}-o_{i}\right)$$

where *s*_i_ is the recorded temperature for the sensor/shield combination for observation *i*,* o*_i_ is the corresponding weather station observation, and *n* is the total number of recorded observations. We also calculated the mean absolute error (MAE) to estimate the overall expected error for each sensor/shield combination:

$$\text{MAE}=\frac{1}{n}\sum_{i=1}^{n}|s_{i}-o_{i}|$$

where the symbols are the same as in the bias equation. Both the bias and the MAE values were calculated separately for the periods from 6 a.m. to 8 p.m. (LST) and 8 p.m. to 6 a.m. to highlight the effects of solar radiation on sensor readings. The error metrics were calculated for each treatment replicate and then averaged to obtain the overall bias or MAE value.

We observed that the Duke Forest permanent weather station may itself be miscalibrated or exposed to site conditions that appear to cause temperature recordings that are biased low in the morning hours around and after sunrise (see Figure 3). While we could not determine the exact cause of these anomalies, it did appear that the bias was consistent across our experimental period. Therefore, we calculated an additional “Adjusted” MAE for the sensor treatment data associated with the Duke Forest site. To calculate the adjusted MAE, we subtracted the daytime MAE of the shielded HOBO Pro sensor at the Lake Wheeler site (the sensor with the lowest overall MAE values) from the MAE of the same sensor at the Duke Forest site. We then subtracted this value, 0.25°C, from the MAE of each sensor/shield combination at the Duke Forest site to obtain an estimated daytime MAE. The rationale for using this adjustment is that we assume that an experiment performed using a permanent weather station of similar quality as to what is available at the Lake Wheeler site should result in similar MAE values for the most accurate sensor/shield treatment (i.e., the shielded HOBO Pro). Therefore, the adjusted MAE for the shielded HOBO Pro at the Duke Forest site equals the MAE for the shielded HOBO Pro at the Lake Wheeler site.

For the impervious surface gradient experiment, we used linear regression to estimate the effect of percent impervious surface (the predictor) on recorded temperatures. Generalized linear regression models were fit independently for each hour of the day using the R function glm (R Core Team, 2016) with assumed Gaussian errors.

We also used linear regression to test the extent to which solar radiation, wind speed, and the interaction between these two covariates, can explain the observed biases in the sensor/shield combinations. For this analysis, we used 2 m solar radiation and wind speed observations from the Lake Wheeler site, which is equivalent to our sensor heights. No solar radiation observations were recorded for the Duke Forest site, so we limited our analysis to the sunny and open Lake Wheeler site (where the effects were expected to be larger). We tested five models (Table 1) that represented variations on the hypothesis that high amounts of solar radiation combined with low wind speeds would result in the largest sensor biases. Once again we used the glm function in R to estimate the regression model, and Akaike Information Criterion (AIC) was used to evaluate model fit and parsimony. Finally, the predicted bias given the interaction between wind speed and solar radiation levels is plotted using the effects package in R (Fox, 2003).

**Table 1:** List of models considered in the regression analysis of the effects of solar radiation and wind speed on air temperature sensor bias relative to the Lake Wheeler weather station

Model #	Model description
1	Solar radiation + error
2	Model 1 + inverse(wind speed)
3	Model 1 + log(inverse[wind speed])
4	Model 2 + solar radiation × inverse(wind speed)
5	Model 3 + solar radiation × log(inverse[wind speed])

Our initial analysis of the recorded temperatures at the Lake Wheeler and Duke Forest sites suggested that several of the iButton treatments were recording nearly identical temperatures (cf. Figure 3). An ANOVA test was conducted and the results (not shown) indicated no statistically significant differences between the four plastic cup treatments at either of the weather station locations, nor were there statistically significant differences between the original “Radshield” and our modified “Small Radshield.” Based on these results, for the regression analysis of solar radiation and wind speed effects (and for the conditional quantile plots in Figure 4), we pooled the recorded temperatures from these groups, increasing the number of replicates, so that the original seven shielded treatments applied to the iButton sensors were reduced to three groups: CUPS (includes the “Cup,” “Cup ventilated,” “Cup, ventilated & sheltered,” and “Cup, ventilated, sheltered, shielded beneath” treatments), Alternative radiation shield (one treatment only), and Radshields (“Radshield,” “Small Radshield”).

All data associated with this research project and manuscript are publicly available and can be found at https://doi.org/10.5066/f7b56hpw. Please contact the first author for any questions concerning the data or metadata.

## 3 RESULTS

### 3.1 Current practice in ecology

**Figure 2:**
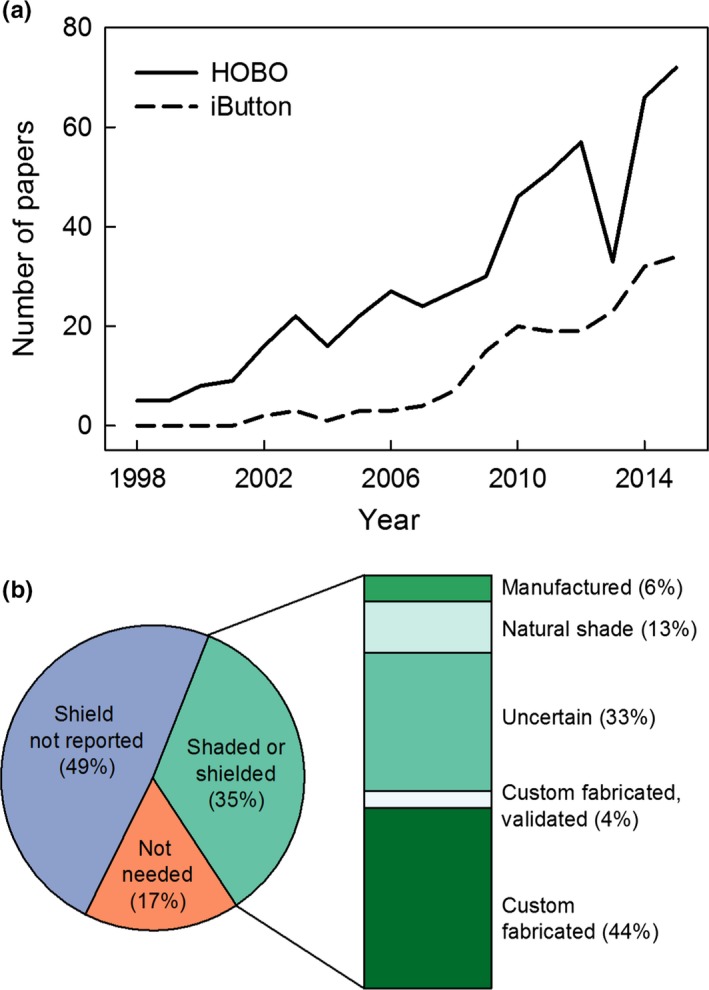
Use and reported radiation shielding practices of iButton and HOBO temperature sensors in ecological field studies. Despite the increasing use of such sensors over time (a), air temperature data collected with these devices are marked by uneven deployment and reporting of methods (b) (*n* = 138 papers that measured air temperature)

Our search of 18 years of literature (1998–2015) in 20 target ecology journals identified a total of 185 papers that used iButtons and 539 papers that used HOBO data sensors. Use of these small, portable sensors has increased over time, from 0 iButton and 5 HOBO papers in 1998 to 34 iButton and 72 HOBO papers in 2015 (Figure 2a). Among the 334 studies that we examined in greater detail, we recorded 417 sensor applications (some studies recorded multiple environmental parameters, and six used both iButtons and HOBOs, resulting in multiple applications per paper.) About one‐third of the applications used sensors to measure and record air temperature. Others used them to record parameters such as body, soil, surface, or water temperature (Table S4).

Among the 138 papers that used the sensors to record air temperature, nearly half did not report radiation shielding practices (Figure 2b). Even if best practices were actually used in these studies, they were not reported in the methods nor illustrated in a figure. Seventeen percent apparently did not require shielding because sensors were deployed at night or indoors, or recorded temperature minima. Only 35% of papers mentioned shielding, and among this subset of 48 papers, only 10% provided either product information for a manufactured shield or validation of a custom‐fabricated shield. In this case, validation could include additional data that in some way assess the efficacy of the shield, or a citation that included such data. The remaining 90% of the papers that mentioned shielding fell into three categories: they used custom‐fabricated shields without validation (44%), provided too little information to understand how sensors were shielded (33%), or deployed sensors in natural shade (13%).

### 3.2 Sensor accuracy under different shield types

Our comparison of different sensor and radiation shield combinations showed large biases across all the custom‐fabricated shields except for the previously published Holden et al. (2013) method and our modification of it (Table 2). Mean biases were largest at the Lake Wheeler site and ranged from a very small positive bias of 0.05°C for the HOBO Pro sensor with the manufactured radiation shield (sensor shown in Figure 1a), to a 3.39°C positive bias for the unshielded HOBO Pendant sensor. The largest bias for a shielded sensor was 3.03°C for the Alternative radiation shield (seen in Figure 1c), while the largest bias among the unpublished methods for custom‐fabricated shields was 2.84°C for the ventilated and sheltered plastic cup (Figure 1g). The smallest bias among the custom‐fabricated methods was 0.75°C for the Radshield design (Figure 1b) described by Holden et al. (2013). Our smaller version of this design (Figure 1e) had a similar bias of 0.81°C. The results for the Duke Forest site followed a similar pattern but with smaller positive biases. Even at this forested site, all iButton and HOBO Pendant sensor/shield combinations (including no shields), with the exception of the Radshield and Small Radshield designs, had positive biases >1°C during the day.

**Table 2:** Mean daytime (6 a.m.–8 p.m. LST) bias for each sensor and shield treatment at the open Lake Wheeler and forested Duke Forest sites

Sensor	Treatment	Daytime bias (°C)	
		Open site	Forested site
HOBO Pro	Manufactured (Gill) shield	0.05	0.34
iButton	Radshield	0.75	0.93
iButton	Small Radshield	0.81	0.93
HOBO Pendant	Radshield	0.92	0.90
HOBO Pro	No shield	1.05	0.72
iButton	No shield	2.17	1.49
iButton	Cup	2.44	1.56
iButton	Cup, ventilated, sheltered, and shielded beneath	2.57	1.80
iButton	Cup, ventilated	2.60	1.74
iButton	Cup, ventilated and sheltered	2.84	1.72
iButton	Alternative radiation shield	3.03	1.71
HOBO Pendant	No shield	3.39	1.51

Sensor/shield combinations are rank‐ordered from lowest to highest bias at the Lake Wheeler site.

The daytime MAE values for most of the sensor/shield combinations were similar to the bias values and had the same rank‐order at the Lake Wheeler site (Table 3). At this site, the largest absolute difference between the bias and the MAE for a sensor was 0.16°C for the shielded HOBO Pro sensor (equal to an MAE of 0.21°C). This indicates that for the rest of the sensor/shield combinations, most of the MAE is explained by the positive bias errors, while the shielded HOBO Pro is accurate to approximately 0.2°C with a small bias of 0.05°C. In contrast, the nighttime MAE values are similar for all sensor/shield combinations, ranging from 0.19°C for the unshielded iButton to 0.30 for the unshielded HOBO Pro at the Lake Wheeler site (Table S5).

**Table 3:** Daytime mean absolute error (MAE) of the comparison of temperatures recorded by each sensor‐shield combination to the weather station standard at open and forested sites

Sensor	Treatment	Daytime MAE (°C)		
		Open site	Forested site	Adjusted forested site
HOBO Pro	Manufactured (Gill) shield	0.21	0.47	0.21
iButton	Radshield	0.75	0.96	0.70
iButton	Small Radshield	0.81	0.98	0.73
HOBO Pendant	Radshield	0.92	0.93	0.67
HOBO Pro	No shield	1.13	0.83	0.57
iButton	No shield	2.18	1.56	1.31
iButton	Cup	2.47	1.63	1.37
iButton	Cup, ventilated, sheltered, and shielded beneath	2.58	1.83	1.58
iButton	Cup, ventilated	2.62	1.80	1.55
iButton	Cup, ventilated and sheltered	2.86	1.76	1.51
iButton	Alternative radiation shield	3.04	1.78	1.52
HOBO Pendant	No shield	3.40	1.58	1.32

An “Adjusted” MAE was calculated for the forested site, by calculating the bias between the HOBO Pro Gill shield and the RAFES station, and subtracting that bias from the original “Forested site” MAE. Sensor‐shield combinations are ordered from smallest (best) to largest MAE at the open site.

We observed a similar rank‐order but with smaller (adjusted) MAE values for the Duke Forest site. The shaded site conditions likely reduce the biases and errors that result from direct solar radiation exposure. However, even in these forested conditions, except for the shielded HOBO Pro sensor, all of the sensor/shield combinations have adjusted MAE values above 0.5°C, and only the manufactured Gill Shield and Radshield designs had MAE values that were <1°C.

**Figure 3:**
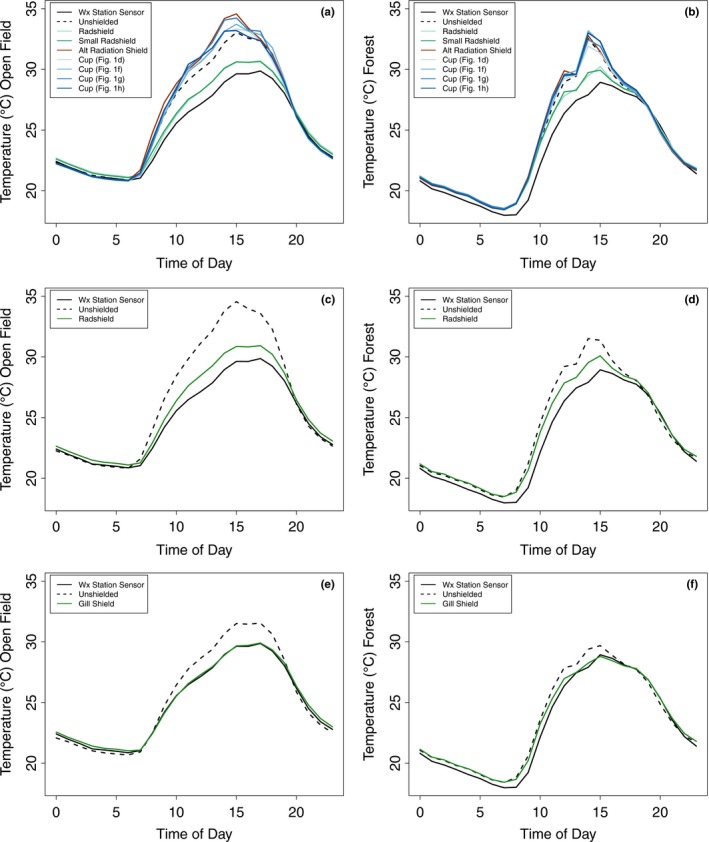
Hourly average temperatures recorded by the weather station sensor (solid black lines) and each combination sensor/treatment at the open Lake Wheeler site (a,c,e) and the forested Duke Forest site (b,d,f). (a,b) Show results for the iButton shield treatments, (c,d) for the Hobo Pendant treatments, and (e,f) for the Hobo Pro treatments. Measurements were recorded from 6–21 and 21–28 August, 2015 at the Lake Wheeler and Duke Forest sites, respectively

These large biases and errors can be seen in the diurnal temperature observations at each site (Figure 3). For some sensor/shield combinations, average midday (between 1100 and 1700 hours LST) readings were more than 5°C warmer, particularly at the sunny Lake Wheeler site. The non‐Radshield iButton treatments show particularly large daytime biases (Figure 3a,b). And consistent with expectations, the unshielded sensors in all cases and at both sites had average midday biases of at least 1°C. Overnight temperature readings were very similar to the observed weather station values, further suggesting that many custom‐fabricated shield types are not adequately mitigating the effects of solar radiation on the accuracy of inexpensive temperature sensors. Notably, the shielded HOBO Pro sensor recorded almost exactly the same temperatures as the Lake Wheeler permanent weather station (Figure 3e). Overall, the majority of the daytime (06:00–20:00 hours LST) observations for all unshielded sensors and custom‐fabricated shielded sensors had biases >1°C, with the four “Cup” treatments and the Alternative radiation shield designs approaching 100% of midday observations that were at least 1°C warmer than the permanent weather stations (Fig. S3).

**Figure 4:**
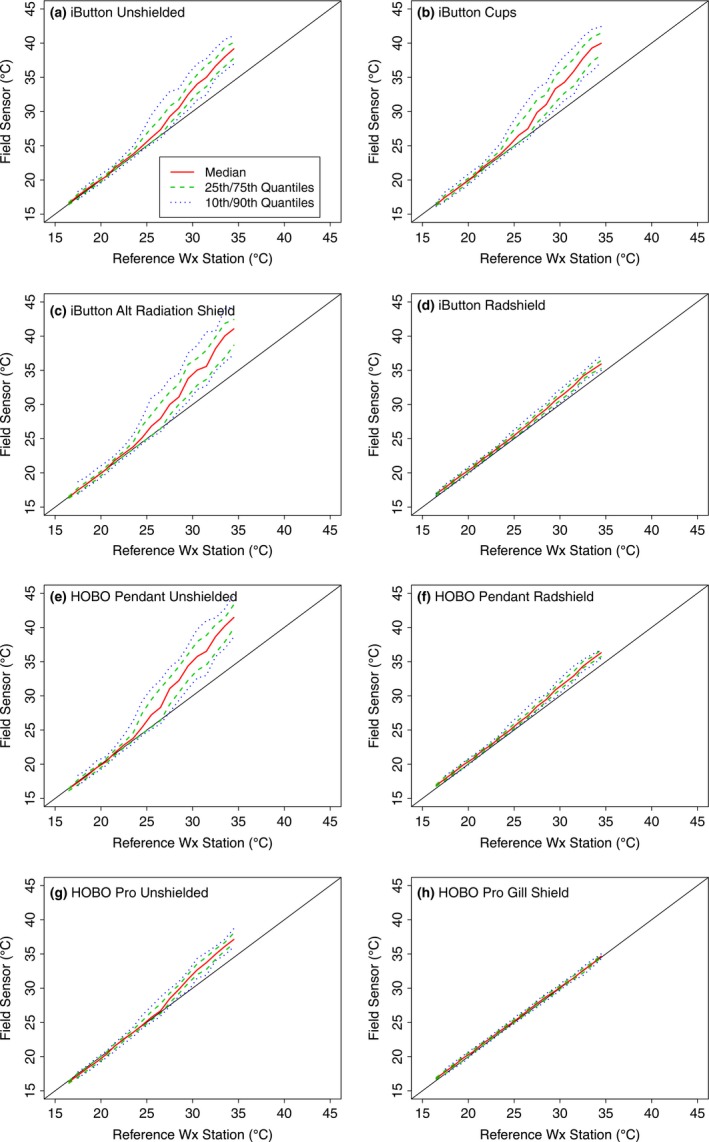
Conditional quantile plots for each sensor/treatment combination at the Lake Wheeler site. Colored lines represent the 50th (red), 25th and 75th (green), and 10th and 90th (blue) quantiles of the recorded sensor temperatures relative to the recorded temperature of the weather station

The effects of solar radiation on sensor bias are even stronger at higher temperatures as seen in the conditional quantile plots for the Lake Wheeler results in Figure 4. Not only is the average bias of the unshielded and custom‐fabricated shield treatments highest during the daytime when solar radiation is strongest, but the variance of the bias increases substantially at the highest temperatures. In particular, the unshielded iButtons (Figure 4a), the unshielded HOBO Pendant (Figure 4e), the combined iButton CUPS treatments (Figure 4b), and the iButton Alternative radiation shield (Figure 4c) all record increasingly extreme temperatures in conjunction with the warmest weather station observations. For some shield/sensor combinations (i.e., Figure 4b,c,e) the 10th and even the 25th quantiles extend well above 40°C when the weather station air temperature readings approach 35°C. In contrast, the biases for the Radshield treatments are more muted (Figure 4d,f), and the recorded temperatures for the shielded HOBO Pro are nearly identical to the permanent weather station observations.

### 3.3 Variation in sensor performance along an impervious surface gradient

**Figure 5:**
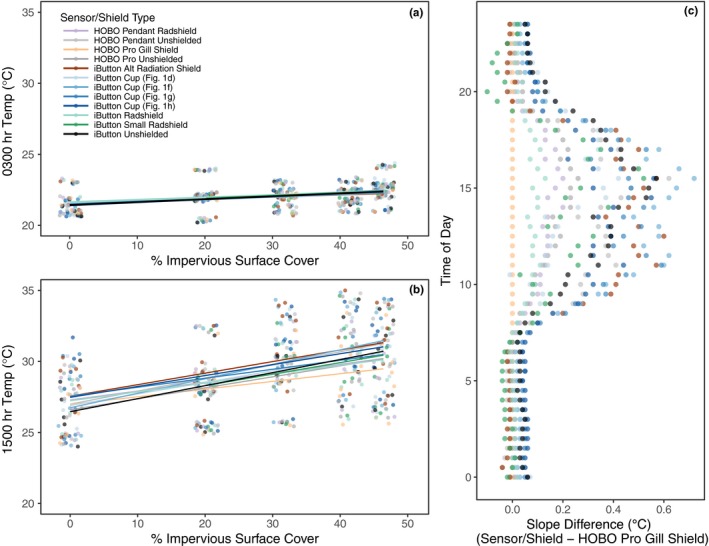
Temperature vs. impervious surface cover at 03:00 hours (a) and 15:00 hours (b) with the least squares fit for each sensor/shield combination. As impervious surface cover increases, biased shields amplify the effect on recorded temperatures. (c) Shows the difference between the estimated slopes (change in temperature per 10% increase in impervious surface cover) of each sensor and the shielded HOBO Pro (the best performing field sensor) for all hours of the day. Orange dots therefore act as a reference line, as they represent the difference between the shielded HOBO Pro and itself (i.e. zero)

As expected, due to urban heat island effects all sensors recorded increasing temperatures with increased impervious surface cover, with stronger effects during daylight hours (Figure 5). However, the variation in observed air temperature during the daytime hours (Figure 5b) was also affected by the type of sensor and shield used. For example, the regression coefficients (with standard errors) that estimate the effect of impervious surface cover on air temperature observations recorded at 3 a.m. all overlap each other (Fig. S4). But the coefficients for the same model fit to the 3 p.m. data show between two and seven nonoverlapping estimates measured against all pairwise combinations of sensors/shields (Fig. S4). Model results indicate that impervious surface cover had the smallest afternoon effect on the shielded HOBO Pro sensor (0.05°C per % impervious surface), while the largest effect was more than double for the iButton with the custom‐fabricated cup depicted in Figure 1f (0.12°C per % impervious surface).

Figure 5c shows all hourly differences between the estimated regression coefficients for each sensor/shield combination and the shielded HOBO Pro sensor (the best performing sensor based on the results from the Lake Wheeler and Duke Forest experiments). Differences are in units of degree celsius per 10% increase in impervious surface cover. Rapid increases in sensors differences, particularly among the custom‐fabricated shields attached to inexpensive sensors, are seen in the morning hours with an initial peak around local solar noon; followed by a late afternoon peak difference that is likely related to maximum daytime heating and upward heat flux. These differences suggest that for some custom‐fabricated sensor/shield combinations, the mean afternoon recorded temperatures over sites with ~50% impervious surface cover could be over 3°C warmer than the shielded HOBO Pro.

### 3.4 Effects of solar radiation and wind speed on sensor performance

**Figure 6:**
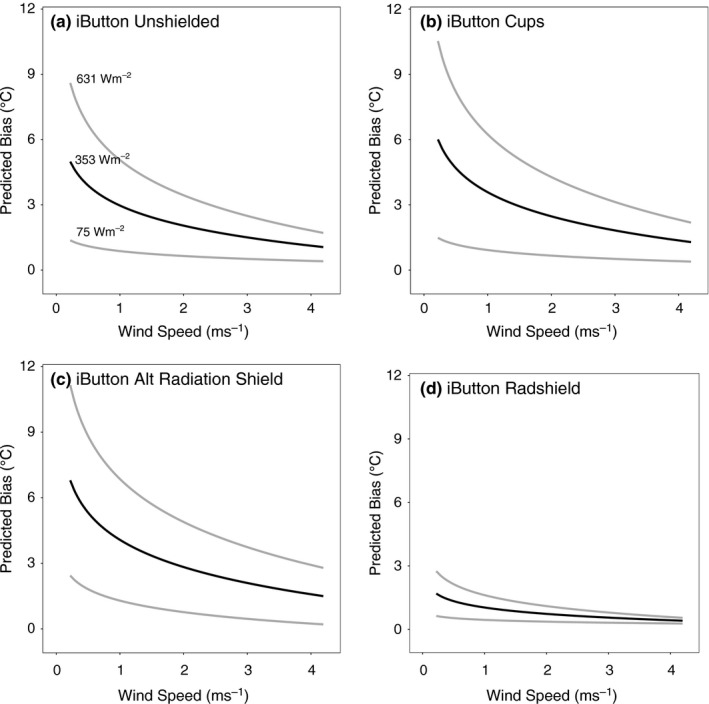
Predicted daytime iButton sensor bias resulting from the interaction of solar radiation and the inverse and log‐transformed wind speed. Heavy black line represents the predicted bias for the mean daytime solar radiation experienced over the experiment period at the Lake Wheeler site. Grey lines show predicted bias at one standard deviation above and below the mean solar radiation. Results are displayed with wind speed backtransformed to the original units of ms^‐1^

**Figure 7:**
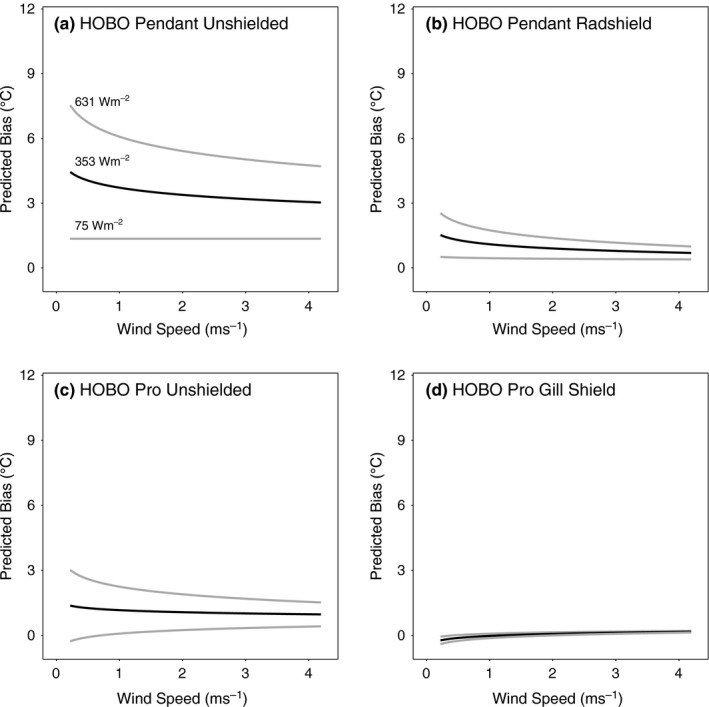
Same as Figure 6 but for the HOBO Pendant and HOBO Pro sensors

To better understand which environmental conditions are most conducive to creating large biases in the sensor/shield combinations, we tested five linear regression models with additive and multiplicative combinations of solar radiation and (inverse) wind speed using the Lake Wheeler air temperature bias results (as described in the 2 section). Models 4 and 5, the two models that included an interaction term between solar radiation and inverse wind speed, had the lowest AIC scores for all eight sensor/shield combinations (Table 4). The results from Model 5, which included as a predictor the log‐transform of inverse wind speed, are shown in Figures 6 and 7, and the results from Model 4 with the absolute wind speed are shown inFigs S5 and S6. These plots show the predicted bias for a given wind speed under three levels of solar radiation (i.e., the mean and ± one standard deviation) at the Lake Wheeler site. Because of the log‐transform of the inverse wind speed predictor, Model 5 is more conservative than Model 4 (in terms of the predicted bias at low wind speeds), although given the very similar AIC values, neither can be ruled out as implausible, and other techniques such as Bayesian model averaging could be used to incorporate information from the full model set (e.g., Terando et al., 2016).

**Table 4:** Results of regression analysis of effect of solar radiation and wind speed on air temperature sensor bias

Model #	Model description	Frequency of lowest AIC score (max = 8)	*R* ^2^ average and (range)
1	Solar radiation + error	0	0.49 (0.05, 0.63)
2	Model 1 + inverse(wind speed)	0	0.51 (0.09, 0.64)
3	Model 1 + log(inverse[wind speed])	0	0.52 (0.09, 0.66)
4	Model 2 + solar radiation × inverse(wind speed)	4	0.54 (0.11, 0.71)
5	Model 3 + solar radiation × log(inverse[wind speed])	4	0.54 (0.09, 0.71)

Results are summarized in terms of the frequency that each tested model had the lowest AIC value for a treatment (*n* = 8 sensor/shield combinations), and the mean and range of the adjusted *R* ^2^ value across those treatments.

Overall, the results in Figures 6 and 7 indicate that large biases could result under conditions of low wind speeds and high solar radiation for most combinations of custom‐fabricated shields with inexpensive temperature sensors. Indeed, for some treatments the predicted low wind speed/high solar radiation biases exceed 10°C (e.g., Figure 6c). In contrast, the predicted shielded HOBO Pro sensor biases are low throughout the range of wind speeds and solar radiation values. The nonlinear nature of these results for the inexpensive shield/sensor combinations suggests that simple, constant bias‐correction methods may not be adequate to control for this error.

## 4 DISCUSSION

Our results definitively show that the accuracy of reported temperature observations in field ecology is highly sensitive to the choice of instruments, materials, and methods used to collect the data. Combined with our review of the literature, this reveals that the quality of reported temperature data likely varies widely across ecological studies. In addition, we found that to collect accurate air temperature data, ecologists must always use high‐quality radiation shields; within our subset of shield types, the manufactured Gill shield was most effective while the two variations on the Radshield performed best among the custom‐fabricated shields. Many custom‐fabricated shields did not prevent biases from being introduced by solar radiation and in some cases, resulted in larger biases than the original unshielded sensors. Therefore, such ad hoc methods must be tested before use in the field. HOBO Pro temperature sensors were by far the most accurate sensors across habitats. These also happen to be the most expensive instruments in our test set (at ~5–40 times the cost of the inexpensive sensors), and therefore, it is unrealistic to expect their use in all studies.

Rather, we caution ecologists to understand the biases associated with their sensors and radiation shields, through their own experiments or through the literature, and take these biases into account when designing experiments and analyses. For example, if measuring maximum temperatures during the day is an objective of a given study, investing in higher quality sensors and shields may be necessary, particularly in exposed environments. We found that for the sunny Lake Wheeler site, inexpensive and improperly shielded sensors had large positive biases in the recorded maximum daily temperatures (Fig. S7). Only the data loggers with the Radshield treatments had mean biases <2°C, while mean biases associated with other shields were >5°C. Conversely, no sensors had mean minimum temperature biases >0.5°C, suggesting that studies that focus on measuring nighttime temperatures may not require investment in the most expensive sensors. Regardless, awareness of biases introduced by the choice of sensor‐shield combination would result in more accurate air temperature data, and thus data that are more readily comparable across studies.

Temperature readings were variable across all environments tested. Compared to weather stations, sensors in the open site had higher temperature recordings for a longer period of time than the sensors in the forested location. The strong and possibly nonlinear interaction between low wind speeds and high solar radiation at the open site is likely to lead to significant biases that could extend to even the best performing radiation shields when applied to low‐cost sensors (e.g., Figure 6d). Under low wind speed and high solar radiation conditions, the accuracy of these data loggers, especially when improperly shielded, is likely reduced due to heating of both the sensor and shield housing. These combined heating effects are seen in Fig. S7, where the recorded maximum temperatures of some shielded iButtons were 0.27–1.91°C higher than the unshielded iButton, while the two Radshields lowered the estimated iButton bias by 3.06 and 3.28°C. In the absence of wind speeds that can efficiently transport heated air molecules away from the sensor, the near‐sensor air temperature will rise above ambient conditions.

Solar heating of the inexpensive sensors/shields is reduced at the forested site, and so the recorded temperatures will be more similar to the surrounding near‐surface environment (Lundquist & Huggett, 2008). However, while trees did dampen some of the temperature extremes, our data indicate that natural shade (although a frequently reported shielding method in the literature) does not completely mitigate solar radiation effects. Furthermore, our impervious surface experiment results show that temperature sensors are affected not only by direct, incoming solar radiation, but also by upward‐directed radiation fluxes from the surface. This is likely to introduce another source of bias in study sites located in urban areas and other environments where radiative heating of improperly shielded sensors can occur due to reflective surfaces or low sun angles, such as in high‐latitude field sites or areas with high albedo values (Huwald et al., 2009).

Temperatures recorded by the inexpensive sensors using Radshields had the lowest biases among the custom‐fabricated shields and were closest to those of the shielded HOBO Pro in the impervious surface gradient experiment. This suggests that this design (and our slight modification of it) is currently one of the best performing custom‐fabricated shields in the literature and may allow for the use of inexpensive temperature sensors across a range of future ecological studies. However, even the Radshield designs resulted in temperature biases that were >0.5°C, and other custom‐fabricated shields may perform similarly. For example, radiation shields constructed out of PVC caps have been used with iButtons in prior studies, and the reported biases were 1°C or less (Ashcroft & Gollan, 2011), while a “homemade Gill shield” tested by Tarara and Hoheisel (2007) also performed reasonably well. Finally, nighttime temperatures in both experiments were least biased and so unshielded sensors may work well when there is little or no direct radiative heating.

Our field results illustrate that designing a small, effective, custom‐fabricated radiation shield is not straightforward, and our literature review suggests that this challenge may be under‐appreciated in the ecological literature. Field ecologists, as a community, do appear to understand that solar radiation affects the accuracy of air temperature measurements. More than one‐third of the papers we examined clearly acknowledged the need for shielding, but the descriptions of the constructed devices often precluded an evaluation of their accuracy in the field. All seven of our iButton shield designs, which had many elements in common with other custom‐fabricated shields mentioned in the literature, yielded biased daytime temperature measurements. Biased temperature data need not invalidate observed patterns or conclusions in the studies that contain them; for example, these measurements may still correctly array sites on an axis from cooler to warmer (Figure 5), but the actual temperature values recorded are not likely to be comparable among studies. About half of the papers we examined did not mention shielding at all, but we suspect that at least some of these authors did not report the type of radiation shields used because they took them for granted, rather than because they did not use them. When manufacturer‐recommended shields are available and habitually deployed, they may appear to be a “package deal” with the sensors themselves. Even this optimistic interpretation points to a widespread need for more thorough reporting to improve repeatability and ensure that best practices are understood by students and readers.

Together, lack of accuracy and standardization in data collection across ecological studies limits the utility of the reported temperature data. We fully acknowledge that our experiment was conducted under a relatively narrow range of environmental and temporal conditions, and that the expected temperature biases will vary by location and climate. Yet, more careful consideration of biases >1°C is likely warranted. For example, biases of 2°C or greater (found in seven of our twelve sensor/shield treatments) equate to the projected annual mean temperature increases over most of North America by the middle of the 21st century due to anthropogenic climate change (Collins et al., 2013). As the climate warms, having accurate temperature observations will be critical for understanding the cascading effects of global climate change at smaller microclimatic scales, and for evaluating the attendant exposure impacts on organisms inhabiting these environs.

We therefore call for an increased awareness among field ecologists of the biases they may introduce when using small, portable temperature sensors without radiation shields, or when using ad hoc methods to construct radiation shields. The increased use of such sensors over the past two decades demonstrates that they meet a demand for collecting climate data on a spatial scale relevant to the organisms’ ecologists study. However, there is a need for more widespread adoption of best practices in their use. We see several ways forward depending on research goals; each of these relies on clear reporting of methods and intentions:

- *Invest in high‐quality aspirated radiation shields, and high‐quality (expensive) sensors when needed*—When actual temperatures are of interest for comparison to other data sources, manufactured shields, or thoroughly validated custom‐fabricated shields that allow for (i) the free flow of air around the sensor, (ii) minimal sensor exposure to solar radiation, and (iii) minimal radiation absorption by the shield (Huwald et al., 2009; Richardson et al., 1999), should be used and their specifications clearly reported. In situations where biases in excess of 0.5°C or more are unacceptable, higher quality sensors and shields may be necessary. More frequent use of published calibration methods before deployment (such as sensor water baths as in Toohey, Neal, and Solin (2014) and Mauger, Shaftel, Trammell, Geist, and Bogan (2015)) could also increase confidence in the results from low‐cost data loggers.
- *Provide clear disclaimers about data use and applicability*—In some cases, it may be helpful to explicitly acknowledge that sensors provide a relative temperature measure within the context of the study but are not meant for use in comparison to other studies.
- *Consider local landscape effects on temperature measurement*—As illustrated in our impervious surface cover experiment, solar radiation effects on some sensors could be exacerbated by additional surface heating. As such, employing the best available materials and methods becomes increasingly important in areas that are likely to experience these conditions.
- *Consider alternative sensor technologies*—The iButton is a compact, inexpensive data logger with a silicon‐based temperature sensor, making it an attractive option in many field ecology studies. However, other established temperature sensors, such as thermocouples or thermistors (which are used in the HOBO Pro data logger), are also available for use with compact data loggers. Ecologists should consider these when evaluating the tradeoffs between time, labor, and cost of deploying any type of temperature sensor in the field.

If temperature data were collected using standard, reliable methods, they could be analyzed across studies, increasing ecologists’ ability to infer climate sensitivity and exposure risks. Careful application of these methods would help to fully realize the opportunity presented by the availability of inexpensive sensors; potentially transforming the field of global change biology by allowing unprecedented comparisons of biotic responses across phylogenetic, spatial, and temporal scales. As field ecology proceeds in the context of rapid anthropogenic climate change, researchers increasingly place their work in a thermal context. This trend has great potential to improve understanding of organismal and ecosystem responses to changing temperatures worldwide. To advance along this path, ecologists must ensure that potential biases in their data are minimized or clearly conveyed. Therefore, we argue that standardizing climate data collection methods in ecology is a critical goal in order to make significant advances in understanding the effects of global change.

## CONFLICT OF INTEREST

None declared.

## AUTHORS CONTRIBUTION

A.J.T. designed and performed experiments, analyzed data, and wrote the paper; E.Y. designed and performed experiments, conducted the literature review, and wrote the paper; E.K.M. and S.G.P. designed and performed experiments and wrote the paper.
