# Supplementary material for: Ad hoc instrumentation methods in ecological studies produce highly biased temperature measurements
Source: Ecol Evol. 2017 Oct 20;7(23):9890–904. doi: 10.1002/ece3.3499 (PMC5723608; doi:10.1002/ece3.3499)
Supplement: Supplementary file 1 [file ECE3-7-9890-s001.doc]

**Supporting Information**

Ad hoc instrumentation methods in ecological studies produce highly biased temperature measurements.

Adam J. Terando, Elsa Youngsteadt, Emily Meineke, and Sara G. Prado

**Table S1.** Number of papers using HOBO and iButton data loggers in 20 ecology journals.

| **Journal** | **Year** | | | | | | | | | | | | | | | | | |  |
| --- | --- | --- | --- | --- | --- | --- | --- | --- | --- | --- | --- | --- | --- | --- | --- | --- | --- | --- | --- |
| **HOBO Studies** | 1998 | 1999 | 2000 | 2001 | 2002 | 2003 | 2004 | 2005 | 2006 | 2007 | 2008 | 2009 | 2010 | 2011 | 2012 | 2013 | 2014 | 2015 | |
| Agriculture Ecosystems & Environment | 0 | 0 | 0 | 0 | 0 | 0 | 0 | 1 | 0 | 1 | 0 | 1 | 4 | 1 | 3 | 5 | 1 | 8 | |
| American Naturalist | 0 | 1 | 0 | 0 | 1 | 0 | 0 | 0 | 0 | 0 | 1 | 0 | 1 | 0 | 1 | 1 | 0 | 4 | |
| Biological Conservation | 0 | 1 | 0 | 0 | 0 | 1 | 2 | 1 | 1 | 1 | 2 | 1 | 3 | 2 | 2 | 2 | 2 | 3 | |
| Conservation Biology | 1 | 0 | 1 | 0 | 1 | 1 | 1 | 0 | 0 | 1 | 1 | 1 | 1 | 1 | 1 | 0 | 2 | 0 | |
| Diversity and Distributions | 0 | 0 | 0 | 0 | 0 | 0 | 0 | 0 | 0 | 0 | 3 | 1 | 0 | 0 | 1 | 0 | 0 | 2 | |
| Ecography | 0 | 0 | 1 | 0 | 0 | 0 | 0 | 0 | 1 | 0 | 0 | 0 | 1 | 0 | 2 | 0 | 0 | 1 | |
| Ecological Applications | 1 | 0 | 0 | 3 | 0 | 0 | 2 | 4 | 6 | 1 | 1 | 3 | 1 | 6 | 4 | 1 | 4 | 4 | |
| Ecological Monographs | 0 | 0 | 0 | 0 | 1 | 2 | 0 | 0 | 0 | 2 | 0 | 0 | 1 | 2 | 0 | 1 | 1 | 1 | |
| Ecology | 2 | 1 | 1 | 2 | 3 | 1 | 4 | 1 | 3 | 0 | 2 | 2 | 5 | 6 | 5 | 4 | 7 | 11 | |
| Ecology Letters | 0 | 0 | 0 | 0 | 0 | 0 | 1 | 0 | 0 | 1 | 0 | 1 | 0 | 0 | 1 | 1 | 3 | 2 | |
| Ecosystems | 0 | 0 | 0 | 0 | 0 | 3 | 1 | 1 | 1 | 2 | 4 | 4 | 2 | 5 | 5 | 1 | 8 | 7 | |
| Global Change Biology | 0 | 0 | 1 | 0 | 0 | 3 | 4 | 2 | 4 | 4 | 5 | 6 | 8 | 12 | 9 | 6 | 8 | 11 | |
| Global Ecology and Biogeography | 0 | 0 | 0 | 0 | 1 | 0 | 0 | 0 | 0 | 1 | 0 | 0 | 1 | 1 | 0 | 1 | 1 | 0 | |
| Journal of Ecology | 0 | 0 | 1 | 0 | 0 | 0 | 1 | 2 | 1 | 4 | 1 | 2 | 3 | 0 | 3 | 2 | 4 | 4 | |
| Journal of Vegetation Science | 0 | 0 | 0 | 0 | 0 | 2 | 0 | 1 | 0 | 2 | 1 | 4 | 2 | 2 | 4 | 1 | 1 | 2 | |
| Landscape and Urban Planning | 0 | 0 | 0 | 0 | 0 | 0 | 0 | 0 | 0 | 0 | 0 | 0 | 0 | 0 | 0 | 0 | 2 | 4 | |
| Landscape Ecology | 0 | 0 | 0 | 0 | 0 | 0 | 0 | 0 | 0 | 0 | 0 | 1 | 0 | 2 | 0 | 0 | 2 | 0 | |
| Oecologia | 1 | 0 | 3 | 3 | 8 | 7 | 0 | 7 | 7 | 4 | 5 | 3 | 9 | 7 | 15 | 7 | 14 | 6 | |
| Oikos | 0 | 2 | 0 | 1 | 1 | 2 | 0 | 2 | 3 | 0 | 0 | 0 | 1 | 0 | 1 | 0 | 1 | 0 | |
| Proceedings of the Royal Society B | 0 | 0 | 0 | 0 | 0 | 0 | 0 | 0 | 0 | 0 | 1 | 0 | 3 | 4 | 0 | 0 | 5 | 2 | |
| **HOBO Total** | **5** | **5** | **8** | **9** | **16** | **22** | **16** | **22** | **27** | **24** | **27** | **30** | **46** | **51** | **57** | **33** | **66** | **72** | |
| **iButton studies** |  |  |  |  |  |  |  |  |  |  |  |  |  |  |  |  |  |  | |
| Agriculture Ecosystems and Environment | 0 | 0 | 0 | 0 | 0 | 0 | 0 | 0 | 0 | 0 | 0 | 1 | 1 | 0 | 0 | 0 | 1 | 0 | |
| American Naturalist | 0 | 0 | 0 | 0 | 0 | 0 | 0 | 0 | 0 | 0 | 0 | 1 | 0 | 2 | 1 | 0 | 0 | 0 | |
| Biological Conservation | 0 | 0 | 0 | 0 | 0 | 0 | 0 | 0 | 0 | 1 | 1 | 0 | 0 | 2 | 1 | 1 | 4 | 3 | |
| Conservation Biology | 0 | 0 | 0 | 0 | 0 | 0 | 0 | 0 | 0 | 0 | 1 | 0 | 0 | 0 | 1 | 1 | 1 | 0 | |
| Diversity and Distributions | 0 | 0 | 0 | 0 | 0 | 0 | 0 | 0 | 0 | 0 | 0 | 1 | 1 | 0 | 0 | 0 | 1 | 0 | |
| Ecography | 0 | 0 | 0 | 0 | 0 | 0 | 0 | 0 | 0 | 0 | 0 | 0 | 1 | 1 | 0 | 2 | 0 | 0 | |
| Ecological Applications | 0 | 0 | 0 | 0 | 0 | 0 | 0 | 0 | 1 | 1 | 0 | 2 | 1 | 1 | 1 | 2 | 2 | 3 | |
| Ecological Monographs | 0 | 0 | 0 | 0 | 0 | 0 | 0 | 0 | 0 | 0 | 0 | 0 | 0 | 1 | 1 | 0 | 0 | 0 | |
| Ecology | 0 | 0 | 0 | 0 | 1 | 2 | 1 | 1 | 0 | 0 | 1 | 2 | 4 | 2 | 0 | 2 | 3 | 3 | |
| Ecology Letters | 0 | 0 | 0 | 0 | 0 | 0 | 0 | 0 | 0 | 0 | 1 | 1 | 0 | 0 | 1 | 0 | 2 | 1 | |
| Ecosystems | 0 | 0 | 0 | 0 | 0 | 0 | 0 | 0 | 0 | 0 | 0 | 0 | 2 | 1 | 1 | 3 | 2 | 1 | |
| Global Change Biology | 0 | 0 | 0 | 0 | 0 | 0 | 0 | 0 | 0 | 1 | 0 | 3 | 2 | 3 | 2 | 4 | 3 | 6 | |
| Global Ecology and Biogeography | 0 | 0 | 0 | 0 | 0 | 0 | 0 | 0 | 0 | 0 | 0 | 0 | 0 | 0 | 1 | 0 | 0 | 0 | |
| Journal of Ecology | 0 | 0 | 0 | 0 | 0 | 0 | 0 | 0 | 0 | 0 | 0 | 2 | 0 | 0 | 1 | 0 | 2 | 1 | |
| Journal of Vegetation Science | 0 | 0 | 0 | 0 | 0 | 0 | 0 | 0 | 0 | 0 | 0 | 0 | 0 | 1 | 0 | 0 | 1 | 2 | |
| Landscape and Urban Planning | 0 | 0 | 0 | 0 | 0 | 0 | 0 | 0 | 0 | 0 | 0 | 0 | 0 | 0 | 1 | 1 | 1 | 1 | |
| Landscape Ecology | 0 | 0 | 0 | 0 | 0 | 0 | 0 | 0 | 0 | 0 | 1 | 0 | 0 | 0 | 0 | 0 | 0 | 0 | |
| Oecologia | 0 | 0 | 0 | 0 | 1 | 1 | 0 | 1 | 1 | 1 | 2 | 2 | 5 | 2 | 5 | 5 | 6 | 8 | |
| Oikos | 0 | 0 | 0 | 0 | 0 | 0 | 0 | 1 | 1 | 0 | 0 | 0 | 0 | 1 | 0 | 0 | 0 | 2 | |
| Proceedings of the Royal Society B | 0 | 0 | 0 | 0 | 0 | 0 | 0 | 0 | 0 | 0 | 0 | 0 | 3 | 2 | 2 | 2 | 3 | 3 | |
| **iButton Total** | **0** | **0** | **0** | **0** | **2** | **3** | **1** | **3** | **3** | **4** | **7** | **15** | **20** | **19** | **19** | **23** | **32** | **34** | |
| **Grand total** | **5** | **5** | **8** | **9** | **18** | **25** | **17** | **25** | **30** | **28** | **34** | **45** | **66** | **70** | **76** | **56** | **98** | **106** | |

**Table S2.** Product information of the three data sensors used in this study. Information was taken in July 2015.

|  | **Hygrochron iButton - Maxim integrated** | **Hobo Pro** | **Hobo Pendant** |
| --- | --- | --- | --- |
| **Model** | DS1923 | HOBO U23-001 Pro v2 | HOBO 8K Pendant UA-001-08 |
| **Operating Range (temp) in air** | -20°C to +85°C | -40° to 70°C | -20° to 70°C |
| **Operating Range (RH) in air** | 0 to 100% RH | 0-100% RH | NA |
| **Accuracy (temp)** | Better Than ±0.5°C from -10°C to +65°C with Software Correction | ±0.21°C from 0° to 50°C | ± 0.53°C from 0° to 50°C |
| **Accuracy (RH)** | 0.6%RH or 0.04%RH | ±2.5% from 10% to 90% RH (typical), to a maximum of ±3.5% including hysteresis | NA |
| **Resolution** | Not provided | 0.02°C at 25°C | 0.14°C at 25°C |
| **Battery life** | 2048 to 8192 Readings (Configuration Dependent) | 3 years with 1 minute or greater logging interval | 1 year typical, user-replaceable |
| **Memory** | 512 Bytes of General-Purpose Memory Plus 64 Bytes of Calibration Memory | 64K bytes | 8K bytes |
| **Price** | US $70.95/ea for 1K | US $170 + $79 for radiation shield | US $42 |

| **Table S3.** Replicate count for each Sensor/Treatment/Site combination. Note, sensor/treatment replicates at Duke Forest were reduced by one each for Small radshield iButton and unshielded HOBO Pro due to sensor loss. | | | | | | | | | |
| --- | --- | --- | --- | --- | --- | --- | --- | --- | --- |
|  |  | Wx Station Sites | | Impervious Surface Sites (Percent) | | | | | |
| **Sensor** | **Treatments** | Lake Wheeler | Duke Forest | Site 1 (0%) | Site 2 (20%) | | Site 3 (31%) | Site 4 (41%) | Site 5 (46%) |
| iButton | Cup | 5 | 5 | 1 | | 1 | 1 | 1 | 1 |
| iButton | Cup, ventilated | 5 | 5 | 1 | | 1 | 1 | 1 | 1 |
| iButton | Cup, ventilated & sheltered | 5 | 5 | 1 | | 1 | 1 | 1 | 1 |
| iButton | Cup, ventilated, sheltered, shielded beneath | 5 | 5 | 1 | | 1 | 1 | 1 | 1 |
| iButton | Alternative Radiation Shield | 5 | 5 | 1 | | 1 | 1 | 1 | 1 |
| iButton | Radshield | 5 | 5 | 1 | | 1 | 1 | 1 | 1 |
| iButton | Small radshield | 5 | 4 | 1 | | 1 | 1 | 1 | 1 |
| iButton | Unshielded | 5 | 5 | 1 | | 1 | 1 | 1 | 1 |
| HOBO Pendant | Radshields | 5 | 5 | 1 | | 1 | 1 | 1 | 1 |
| HOBO Pendant | Unshielded | 5 | 5 | 1 | | 1 | 1 | 1 | 1 |
| HOBO Pro | Manufactured (Gill Shield) | 5 | 5 | 1 | | 1 | 1 | 1 | 1 |
| HOBO Pro | Unshielded | 5 | 4 | 1 | | 1 | 1 | 1 | 1 |

**Table S4.** Summary of iButton and HOBO applications in the literature

|  | Ibuttons (n = 170) | | Hobos (n = 170) | | All papers (n = 334) | |
| --- | --- | --- | --- | --- | --- | --- |
| Variable | Number | Proportion | Number | Proportion | Number | Proportion |
| Air temperature | 72 | 0.35 | 66 | 0.32 | 138 | 0.33 |
| Humidity | 23 | 0.11 | 26 | 0.12 | 49 | 0.12 |
| Soil temperature | 37 | 0.18 | 39 | 0.19 | 76 | 0.18 |
| Water temperature | 24 | 0.12 | 41 | 0.20 | 65 | 0.16 |
| Other | 52 | 0.25 | 37 | 0.18 | 89 | 0.21 |
| Total applications | 208 |  | 209 |  | 417 |  |

**Table S5. Mean absolute error (MAE) for each sensor/shield treatment for the daytime (6am to 8pm LST) and nighttime (8pm to 6am) periods at the Lake Wheeler site. Sensor/shield combinations are rank-ordered from lowest to highest MAE daytime values.**

| Sensor | Treatment | MAE (°C) | |
| --- | --- | --- | --- |
|  |  | Day | Night |
| HOBO Pro | Manufactured (Gill) shield | 0.21 | 0.20 |
| iButton | Radshield | 0.75 | 0.30 |
| iButton | Small Radshield | 0.81 | 0.23 |
| HOBO Pendant | Radshield | 0.92 | 0.27 |
| HOBO Pro | No shield | 1.13 | 0.30 |
| iButton | No shield | 2.18 | 0.19 |
| iButton | Cup | 2.47 | 0.28 |
| iButton | Cup, ventilated, sheltered & shielded beneath | 2.58 | 0.23 |
| iButton | Cup, ventilated | 2.62 | 0.25 |
| iButton | Cup, ventilated & sheltered | 2.86 | 0.25 |
| iButton | Alternative radiation shield | 3.04 | 0.24 |
| HOBO Pendant | No shield | 3.40 | 0.26 |

**
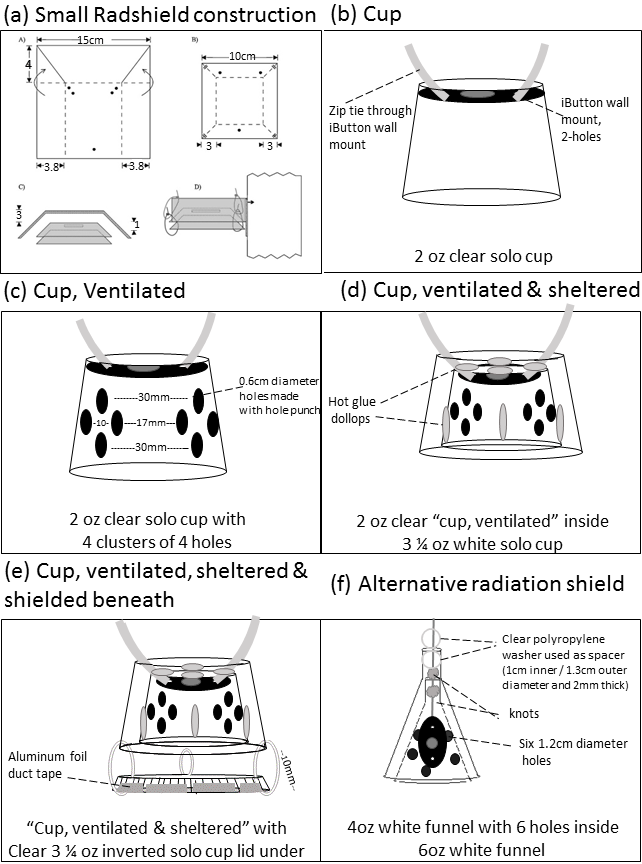
**

**Figure S1.** Construction details for custom-fabricated shields. iButtons and Hobo Pendants were placed in (**a**), whereas only iButtons were used in shields (**b**)-(**f**). All iButtons were placed in an iButton Wall Mount, 2 holes (Embedded Data Systems, Lawrenceburg, KY, USA Part Number #DS9093S). A 30cm long and 0.5cm wide zip tie was used to hang all the Solo® cup shields. (**a**) **Small Radshield** – Image adapted from Fig. 2 in Holden et al. (2013). Measurements were adjusted to be approximately half the size of the original Radshield. Other than the difference in dimensions and the brand of foil tape (we used Nashua® Aluminum Multi-purpose Foil tape 3.2-mil thick, UPC # 033656304505), the construction, assembly methods, and materials are identical to those described in Holden et al. (2013). (**b**) **Cup** – Clear 2oz Solo® cup as described by Meineke et al. (2013) and Carper et al. (2014). (**c**) **Cup, Ventilated** – Clear 2oz Solo® cup with 4 clusters of 4 holes in diamond pattern, made using a hole punch. (**d**) **Cup, Ventilated & Sheltered** – White 3 ¼ oz Solo® cup with “Cup, Ventilated” placed inside. Four strips (~3mm high) of hot glue were placed on the sides, and four dollops (~5mm high) of hot glue were placed on top of the “Cup, Ventilated”, acting as spacers between the two Solo® cups. (**e**) **Cup, Ventilated, Sheltered & Shielded beneath** – Main shield is the same design as “Cup, Ventilated & Sheltered”. An upside-down 3 ¼ oz clear Solo® cup lid with Nashua® Aluminum Multi-purpose Foil tape was used as a shield against incoming reflected radiation from the ground. The lid was tied to the white 3 ¼ oz Solo® cup using a 10cm long and 0.2cm wide zip tie. (**f**) **Alternative radiation shield** designed as described by Hubbart (2011), however a 6oz and 4oz funnel (TheToolWorkshop, Sicklerville, NJ, USA, Item #604368) was used instead of an 8oz and 6oz. One clear polypropylene washer (New Process Fibre, Greenwood, DE, US, Item #WAT3924) was used as a spacer between the two funnel spouts, and one was used at the spout of the 8oz funnel to prevent the iButton from sliding down. Holes in the 4oz funnel were melted out of the plastic using a 1.27cm cork borer heated over a Bunsen burner. A 7-strand electric fence wire was used to hang the Alterative radiation shield.


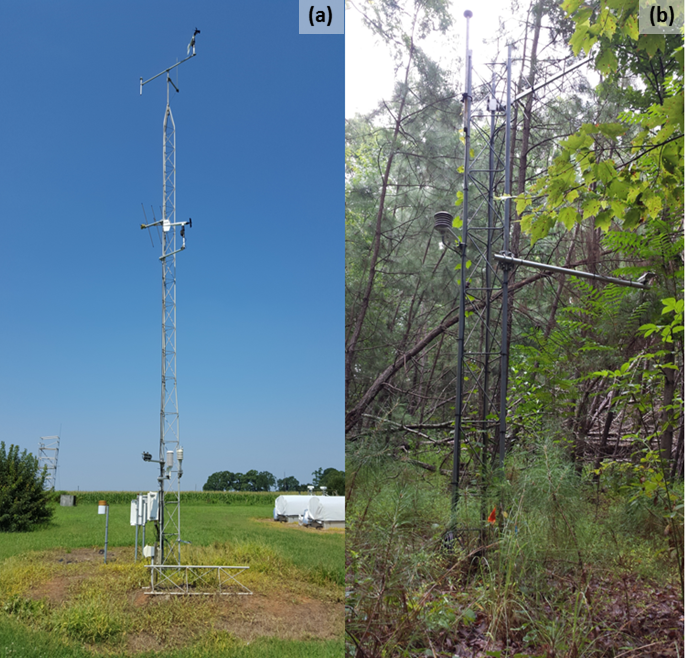


Figure S2. (a) ECONet weather station in full sun. (b) RAFES weather station under a forest canopy (right).

**Figure S3.** Inexpensive temperature sensors will be biased during daylight hours, even in forested conditions. Proportion of all recorded observations with errors of at least 1°C (defined as the absolute difference between the permanent weather station sensor and the tested sensors) at the open Lake Wheeler site ((a), (c), (e)) and the forested Duke Forest site ((b), (d), (f)). (a)-(b) show results for the iButton shield treatments, (c)-(d) for the Hobo Pendant treatments, and (e)-(f) for the HOBO Pro treatments.

β_1_

(0300)

β_2_

(1500)

**Figure S4.** Forest plot of estimated regression coefficients for the linear model of impervious surface cover effects on air temperature measured at 0300 hours and 1500 hours. Dashed vertical lines represent the mean regression coefficient across all the sensor/shield combinations for the two time periods. All twelve combinations had overlapping coefficient estimates for the 0300 hour model, while for the 1500 hour model the number of pairwise overlapping coefficient estimates for each shield/sensor combination with all other shield treatments ranged between 4 (for the HOBO Pro Gill Shield) and 9 (for three of the treatments).

**Figure S5.** Same as Figure 6 but results depict the predicted multiplicative effects of solar radiation and *absolute* (as opposed to logarithmic) wind speed.

**Figure S6.** Same as Fig. S5 but for the HOBO Pendant and HOBO Pro sensor/shield combinations.

**Figure S7.** Boxplots of the differences in the recorded maximum daily temperatures between the tested sensor/shield combinations and the permanent Lake Wheeler weather station. Red boxes show the differences between temperatures recorded by the data logger at the time of recorded maximum daily temperature at the Lake Wheeler weather station. Green boxes show the differences between the maximum daily temperatures recorded by both the weather station and the data loggers. Blue boxes show the differences between the maximum daily temperature recorded by the data logger and the corresponding temperature recorded by the weather station. Mean bias values for the max daily temperatures (green box and whiskers) are listed above each box.
